# Transforming Cancer Diagnostics: The Emergence of Liquid Biopsy and Epigenetic Markers

**DOI:** 10.1002/mco2.70388

**Published:** 2025-09-14

**Authors:** Debalina Saha, Pritam Kanjilal, Mandeep Kaur, Soumya V. Menon, Ayash Ashraf, M. Ravi Kumar, Taha Alqahtani, Shikha Atteri, Daniel Ejim Uti, Bikram Dhara

**Affiliations:** ^1^ Department of Microbiology St. Xavier's College (Autonomous) Kolkata India; ^2^ Department of Sciences Vivekananda Global University Jaipur Rajasthan India; ^3^ Department of Chemistry and Biochemistry School of Sciences, JAIN (Deemed to be University) Bangalore Karnataka India; ^4^ Chandigarh Pharmacy College Chandigarh Group of College, Jhanjeri Mohali Punjab India; ^5^ Department of Chemistry Raghu Engineering College Visakhapatnam Andhra Pradesh India; ^6^ Department of Pharmacology College of Pharmacy, King Khalid University Abha Saudi Arabia; ^7^ Department of Research and Publications Kampala International University Kampala Uganda; ^8^ Department of Biochemistry, Faculty of Basic Medical Sciences Federal University of Health Sciences Otukpo Benue Nigeria; ^9^ Center for Global Health Research Saveetha Institute of Medical and Technical Sciences Chennai India

**Keywords:** cancer diagnostics, DNA methylation, epigenetic markers, liquid biopsy, noncoding RNAs

## Abstract

Liquid biopsy represents a transformative approach in oncology, enabling noninvasive disease detection and monitoring through epigenetic signals in circulating tumor DNA (ctDNA), nucleosomes, and noncoding RNAs. Tumor initiation is driven by epigenetic modifications, including DNA methylation, histone alterations, and dysregulated noncoding RNAs, which disrupt gene regulation, cell cycle control, DNA repair, and metastatic processes. This review systematically examines recent evidence on DNA methylation, histone marks (e.g., H3K27me3, H3K18ac), and noncoding RNAs (miRNAs, lncRNAs) as biomarkers for early cancer detection, prognosis, and therapeutic response. Particular focus is placed on aberrant DNA methylation (e.g., hypermethylation of CDKN2A, RASSF1A) and altered histone modifications (e.g., EZH2‐mediated silencing) as indicators of tumor heterogeneity and evolution. Stable and specific in biofluids, noncoding RNAs such as oncogenic miR‐21, tumor‐suppressive miR‐34a, and metastasis‐associated MALAT1/HOTAIR further enhance clinical applicability. Recent detection methods, including bisulfite sequencing, ChIP‐seq, and RNA‐seq, have advanced biomarker profiling, though challenges remain in standardization and low‐abundance detection. With over 12 active clinical studies validating their utility, integration of epigenetic markers with AI and multiomics holds promise for individualized, dynamically guided oncology care. Future innovations, such as chromatin accessibility analysis and cfDNA fragmentation profiling, may further refine diagnostic precision and therapeutic monitoring.

## Graphical abstract

Liquid biopsy is becoming prominent in cancer diagnostics and management due to its advantage of being noninvasive compared to traditional tissue biopsy. This technique processes blood sample to identify circulating biomarkers like cell‐free DNA, circulating tumor cell, and exosomal RNA. Of these, DNA methylation, histone modifications, and noncoding RNA have been revealed as significant biomarkers for use in cancer diagnosis at early stage, prognosis and prediction of therapeutic responsiveness. When liquid biopsy is combined with epigenetic characterization, tracking of tumor kinetics is possible in real‐time, opening up new possibilities for personalized therapeutic management of malignant disease in oncology. Such synergy will result in improved sensitivity and specificity and a deeper understanding of cancer in general.2

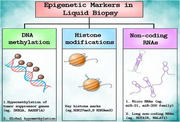

## 1 Introduction

**FIGURE 1:**
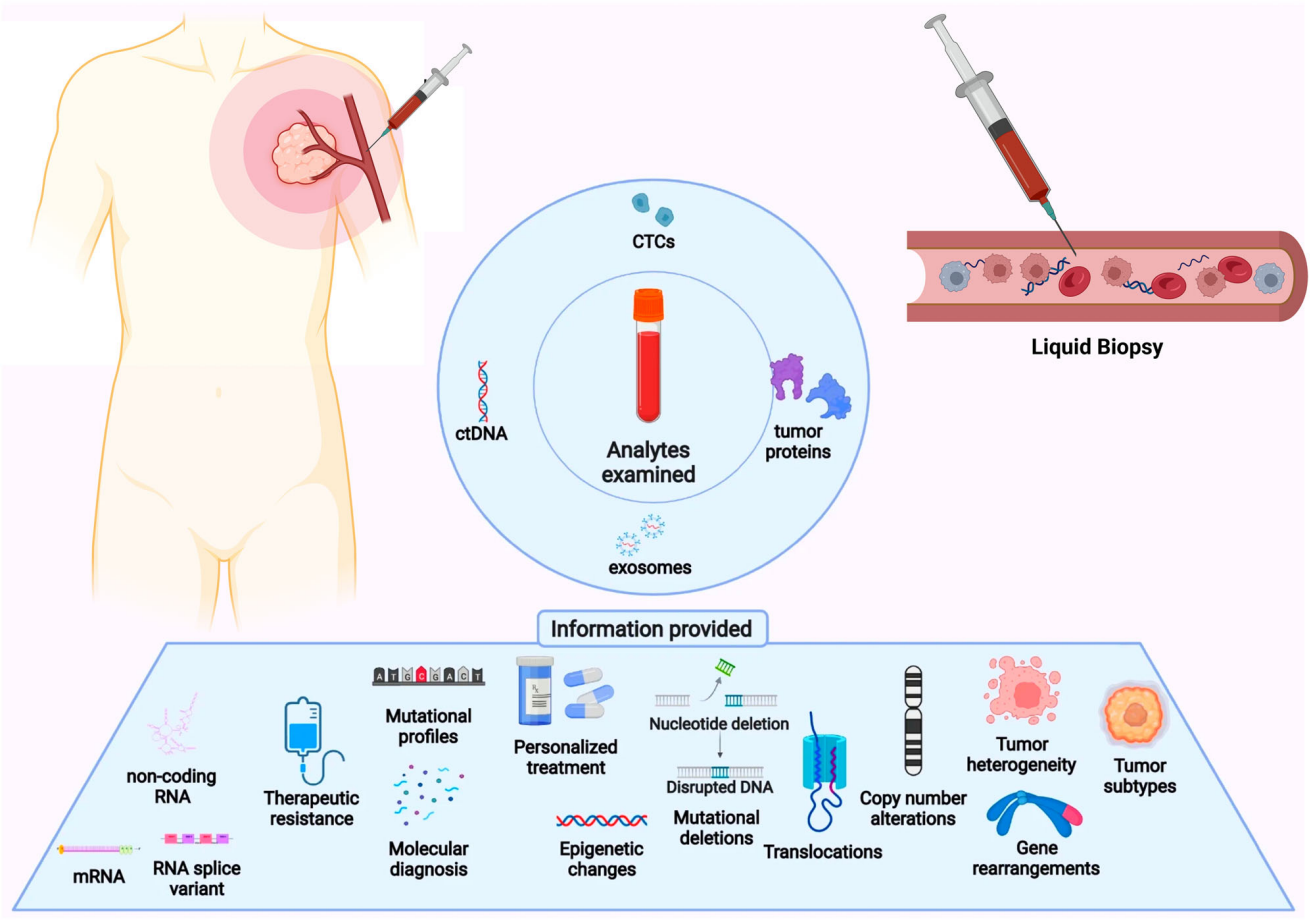
Overview of the analytes examined and diagnostic information provided by liquid biopsy: This figure summarizes the key analytes detectable in liquid biopsy samples, including circulating tumor DNA (ctDNA), circulating tumor cells (CTCs), exosomes, and microRNAs, along with the types of clinically actionable information they provide, such as molecular diagnoses, tumor heterogeneity, and marker‐guided personalized treatment (adapted from ref. [189]. https://doi.org/10.1186/s12943‐022‐01543‐7 by license CC BY 4.0 Copyright 2022 The Authors).

Traditionally, cancer diagnosis depended mainly on tissue biopsy procedures. Although, these techniques were both surgically demanding and restricted to heterogeneous tumor characteristics as well as recurrent examination limitations. Molecular biology research and the discovery of circulating tumor DNA (ctDNA) and other cancer‐derived materials in bodily fluids during the late 20th century allowed liquid biopsy to become more prominent. In recent years, the field of liquid biopsy has emerged as a revolutionary approach in oncology, offering a minimally invasive means to obtain critical molecular insights from cancer patients (Figure 1). This technique focuses on analyzing genetic and epigenetic markers present in bodily fluids such as peripheral blood, urine, saliva, cerebral spinal fluid, and breast milk. Such analyses provide a real‐time snapshot of the heterogeneity and dynamics of tumors, facilitating a thorough assessment of their molecular landscape. Liquid biopsies study circulating tumor cells (CTCs), circulating cell‐free DNA (cfDNA), and other materials shed by tumors, including “tumor‐educated platelets” (TEPs) and vesicles like exosomes. These components not only carry proteins, RNA, and DNA from the primary tumor but also from metastatic sites and the tumor microenvironment, thereby reflecting changes within cancer cells that are critical for understanding disease progression [1, 2, 3, 4].

Among the epigenetic markers explored through liquid biopsy, DNA methylation patterns, histone modifications, and noncoding RNA (ncRNA) profiles are of particular interest due to their profound impact on gene regulation and tumor behavior. Aberrant DNA methylation, such as the hypermethylation of tumor‐suppressor gene promoters (e.g., CDKN2A, RASSF1A), is a well‐recognized hallmark of cancer. These methylation patterns can serve as reliable biomarkers for early detection and prognosis [5, 6]. Similarly, histone modifications, including trimethylation of histone H3 on lysine 27 (H3K27me3) and lysine 9 (H3K9me3), provide insights into changes in chromatin structure and gene accessibility, enriching our understanding of the epigenetic landscape of tumors [7, 8].

ncRNAs, particularly microRNAs (miRNAs) and long noncoding RNAs (lncRNAs), play essential roles in modulating gene expression and are crucial for the development and progression of cancer. These ncRNAs regulate gene expression post‐transcriptionally by targeting mRNA for degradation or translational repression. miRNAs like miR‐21 function as oncogenes by suppressing tumor‐suppressor genes such as PTEN and PDCD4, thereby activating pathways that promote cell proliferation and survival. This is observable in various cancers, including breast, lung, and colorectal cancers. Conversely, miRNAs such as miR‐15 and miR‐16 act as tumor suppressors, and their reduced expression is linked to diseases like chronic lymphocytic leukemia and prostate cancer [3]. lncRNAs such as HOTAIR and MALAT1 affect gene expression through mechanisms like chromatin remodeling and transcriptional interference, which are pivotal in processes such as metastasis and tumor growth [3]. The integration of these molecular signatures through liquid biopsy not only facilitates the early detection and diagnosis of malignancies but also enables continuous monitoring of treatment response and the identification of potential resistance mechanisms. This noninvasive approach, which includes profiling miRNAs and lncRNAs, provides a dynamic view of the tumor's genetic and epigenetic landscape, allowing for tailored therapeutic strategies based on the individual molecular profiles of patients' tumors. Such advancements underscore the potential of liquid biopsies in transforming cancer diagnosis, prognosis, and personalized medicine, making it a cornerstone of modern oncological practices.

This review is structured to examine DNA methylation first as the main epigenetic indicator before discussing histone modifications and ncRNAs. The study details detection methods before exploring current clinical applications and trials followed by directions for future liquid biopsy research. The review systematically illustrates current developments to serve as a guidance system for both clinicians and researchers working on developing epigenetics‐based noninvasive cancer diagnostic approaches.

## 2 DNA Methylation Markers: A New Frontier in Noninvasive Cancer Diagnosis

In the realm of cancer diagnostics, DNA methylation patterns offer a promising avenue for early detection and monitoring through liquid biopsies. This technique involves the analysis of cfDNA extracted from bodily fluids like blood or urine. By examining the methylation patterns present in this cfDNA, clinicians can infer the tissue of origin, indicating the potential presence of cancer cells shedding their DNA into the bloodstream. This approach is particularly valuable for cancers that are difficult to detect early by conventional means, such as pancreatic and ovarian cancers. The identification of tissue‐specific methylation patterns not only helps in diagnosing cancer but also provides insights into the cancer's stage and progression, making it a powerful tool for personalized medicine [9, 10].

### 2.1 Mechanisms and Clinical Applications of DNA Methylation Analysis in Cancer

**FIGURE 2:**
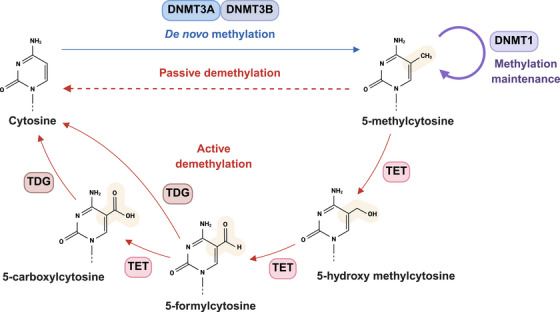
Mechanism of DNA methylation: this schematic summarizes key pathways involved in the establishment, maintenance, and removal of DNA methylation marks—including de novo methylation by DNMT3A/B, maintenance methylation by DNMT1, and active demethylation via TET enzymes and TDG‐mediated base excision repair—illustrating the dynamic regulation of epigenetic states in gene expression control (created with BioRender.com).

DNA methylation, the most extensively studied epigenetic modification, involves the covalent addition of a methyl group (–CH_3_) to the 5‐position carbon of the cytosine pyrimidine ring, predominantly within CpG dinucleotides (Figure 2). These dinucleotides frequently cluster in regions known as CpG islands, which are often located in or near gene promoters and are crucial for the regulation of gene expression [3, 11, 12]. When a methyl group is added to cytosine, it forms 5‐methylcytosine (5mC), which can inhibit gene transcription. This inhibition occurs either by blocking the binding of transcription factors to the DNA or by attracting proteins that compact the chromatin structure, thereby reducing gene expression [13].

In the context of cancer, aberrant patterns of DNA methylation are a hallmark, often leading to the progression and development of the disease. For instance, the hypermethylation of CpG islands within tumor‐suppressor gene promoters can lead to their silencing, effectively disabling key pathways that regulate cell growth and apoptosis. Conversely, global hypomethylation observed in cancer cells can lead to genomic instability and increased tumorigenicity (Table 1) [14].

**TABLE 1:** The critical elements of DNA methylation in the context of cancer.

Aspect of DNA methylation	Description	Implications in cancer and applications in liquid biopsy	References
**Biological mechanism**	DNA methylation involves the addition of a methyl group to the 5‐position of the cytosine ring within CpG dinucleotides, commonly found in CpG islands near gene promoters. **Key details**: Methylation is catalyzed by DNA methyltransferases and results in 5‐methylcytosine (5mC), which can alter gene expression by affecting transcription factor binding or chromatin structure.	**Implications in cancer**: Aberrant methylation can lead to gene silencing or activation, disrupting normal cellular functions and contributing to oncogenesis.	[3]
		**Applications in liquid biopsy**: Detecting abnormal methylation patterns in cfDNA offers insights into the epigenetic alterations associated with tumors.	
**Role in gene regulation**	Methylation at CpG islands typically suppresses gene transcription by preventing transcription factor access or by recruiting methyl‐binding proteins that change chromatin structure to a more closed state. **Key details**: In normal cells, methylation regulates gene expression during development and maintains cellular identity.	**Implications in cancer**: In cancer, hypermethylation of tumor‐suppressor genes or hypomethylation of oncogenes disrupts normal control mechanisms, facilitating uncontrolled cell growth and metastasis.	[4]
		**Applications in liquid biopsy**: Liquid biopsy can identify methylation changes that indicate the presence and type of cancer, aiding in early detection and targeted therapy.	
**Detection techniques**	Methylation‐specific PCR, bisulfite sequencing, and next‐generation sequencing are common methods used to analyze methylation patterns. **Key details**: These techniques vary in sensitivity, specificity, and the ability to quantify methylation levels across the genome.	**Implications in cancer**: Methylation patterns serve as biomarkers for cancer diagnostics, prognostics, and monitoring therapeutic responses.	[11]
		**Applications in liquid biopsy**: Liquid biopsies use these techniques to analyze cfDNA from blood or other body fluids, providing a noninvasive method to monitor tumor dynamics and treatment efficacy	
**Clinical relevance**	The stability of methylated DNA in blood and other bodily fluids makes it an excellent target for liquid biopsy in cancer diagnostics. **Key details**: The assessment of methylation offers a reliable approach to track tumor origin and behavior over time.	**Implications in cancer**: Patterns of DNA methylation can predict tumor behavior, response to drugs, and potential relapse.	[12]
		**Applications in liquid biopsy**: Regular monitoring of cfDNA methylation in patients can guide treatment decisions, adjust therapies in real‐time, and evaluate resistance mechanisms.	

The advent of liquid biopsy has significantly enhanced the capabilities for noninvasive cancer diagnostics by utilizing these methylation patterns as biomarkers. Through the analysis of cfDNA extracted from bodily fluids such as blood, it is possible to detect and analyze cancer‐specific methylation signatures. This powerful technique allows for the early detection of malignancies, monitoring of disease progression, and assessment of responses to therapy, all in real‐time. By providing a detailed snapshot of the tumor's genetic landscape without the need for invasive surgical procedures, liquid biopsy based on DNA methylation analysis not only deepens our understanding of tumor biology but also markedly improves patient management by enabling precise and timely therapeutic interventions based on the epigenetic landscape revealed through these noninvasive methods [3, 4, 11].

### 2.2 DNA Methyltransferases and the Epigenetic Regulation of Cancer Progression

The addition of methyl groups is catalyzed by specific enzymes known as DNA methyltransferases (DNMTs). Among these, DNMT1 is responsible for maintenance methylation, ensuring that the methylation pattern of the parent DNA strand is faithfully copied onto the daughter strand during DNA replication [14]. On the other hand, DNMT3A and DNMT3B, along with others like DNMT3L, facilitate the establishment of new methylation patterns—known as de novo methylation—which are crucial during development and cell differentiation [15, 16, 17]. DNA methylation plays a multifaceted role in the development and progression of cancer, affecting gene expression, genomic stability, and cellular phenotype. As an epigenetic mechanism, it involves the addition of methyl groups to the DNA, typically at cytosine bases within CpG dinucleotides. This modification can have profound implications for cellular function, particularly in the context of cancer, where normal patterns of methylation are often disrupted. Understanding the detailed impact of these changes can provide critical insights into cancer biology and aid in developing targeted diagnostic and therapeutic strategies (Table 2).

**TABLE 2:** Various aspects of DNA methylation in cancer.

Aspect	Description	Implications in cancer	References
**Gene silencing**	Hypermethylation of DNA at gene promoters leads to the silencing of tumor‐suppressor genes. This disruption in gene expression is pivotal in allowing unchecked cell proliferation. **Key genes involved**: CDKN2A (p16, p15)	Loss of tumor‐suppressor function facilitates unchecked cellular proliferation and tumorigenesis, commonly seen in melanomas, gliomas, and certain leukemia's.	[14]
**Genomic instability**	Global hypomethylation, particularly prevalent in repetitive regions and transposable elements, can lead to genomic instability by allowing these elements to become active and insert into new genomic locations. This disrupts genomic integrity and can activate oncogenes. **Key genes involved**: Not specific to single genes	Increased mutation rates and chromosomal instability contribute to aggressive cancer phenotypes and poorer prognosis in cancers like colorectal, liver, and lung cancers.	[5]
**Liquid biopsy biomarkers**	DNA methylation patterns in cell‐free DNA (cfDNA) from bodily fluids can be used as noninvasive biomarkers for cancer detection. These patterns indicate the tissue of origin of cfDNA and help in early cancer detection, monitoring progression, and assessing treatment response. **Key genes involved**: Not specific to single genes	Enhances early cancer detection, particularly for hard‐to‐diagnose cancers such as pancreatic and ovarian cancers, and provides a means for ongoing monitoring of cancer progression and treatment efficacy without the need for invasive procedures.	[3]
**Cellular pathways impact**	Promoter hypermethylation in genes involved in crucial cellular pathways leads to their silencing, affecting cell cycle regulation, DNA repair, apoptosis, and cell adhesion—key processes that when disrupted, contribute to cancer progression and metastasis. **Key genes involved**: CHFR, MGMT, DAPK, Caspase 8, Cadherins	‐**CHFR**: Bypassing cell cycle checkpoints increases genetic aberrations. ‐**MGMT**: Impaired DNA repair contributes to mutation accumulation and chemoresistance. ‐**DAPK and Caspase 8**: Avoiding apoptosis promotes survival of malignant cells. ‐**Cadherins**: Loss of cell adhesion facilitates metastasis.	[2]

### 2.3 Gene Silencing in Cancer Through DNA Methylation

The comprehensive overview underscores the intricate mechanisms through which DNA methylation leads to the silencing of tumor‐suppressor genes in cancer. This process is critical in the epigenetic regulation observed across various cancer types. For instance, enzymes such as DNMTs and EZH2 overexpress in cancerous cells, leading to abnormal methylation of CpG islands at gene promoters, which results in the repression of crucial genes that regulate cellular processes such as the cell cycle, apoptosis, and metastasis. Specifically, the hypermethylation of the CDKN2A gene promoter exemplifies this phenomenon, leading to the inactivation of its gene products, p16 and p15. These proteins play a pivotal role in cell cycle regulation by inhibiting certain cyclin‐dependent kinases, thereby preventing the phosphorylation of the retinoblastoma protein (pRb). This inhibition is essential for maintaining cell cycle integrity, particularly at the G1 to S phase checkpoint, where the cell assesses DNA integrity before proceeding with replication. The silencing of p16 and p15 through hypermethylation results in a loss of this crucial control, causing unchecked cell division and significantly contributing to tumor growth and development [9, 18, 19, 20] (Table 3).

**TABLE 3:** Intricate processes involved in the epigenetic regulation of gene expression in cancer via DNA methylation.

Category		Description	Therapeutic approaches	References
**Mechanisms of DNA methylation**	DNA methylation process	Involves the addition of a methyl group to the 5‐position carbon of the cytosine ring within CpG dinucleotides, leading to the formation of 5‐methylcytosine (5mC). This modification typically occurs at CpG islands near gene promoters, influencing gene expression by blocking transcription factor binding. **Key genes/molecules**: DNMTs, EZH2 **Cancer types affected**: All cancer types	Nucleoside analogues (e.g., Azacitidine, Decitabine), EZH2 inhibitors	[14, 15, 21]
	Role of EZH2 in methylation	EZH2, part of the polycomb repressive complex 2 (PRC2), adds methyl groups to histone proteins, compounding the effects of DNA methylation. Overexpression of EZH2 correlates with the silencing of specific genes involved in tumor suppression and cellular differentiation. **Key genes/molecules**: EZH2, PRC2 **Cancer types affected**: Breast, prostate, others	EZH2 inhibitors (e.g., Tazemetostat)	[14, 15, 22]
**Impact on tumor‐suppressor genes**	Hyper/hypomethylation effects	Hypermethylation of tumor‐suppressor gene promoters leads to their silencing, while global hypomethylation can activate oncogenes or increase genomic instability. **Key genes/molecules**: CDKN2A (p16, p15), RARB **Cancer types affected**: Melanomas, gliomas, leukemia, various solid tumors	DNMT inhibitors, combination therapies with HDAC inhibitors and targeted therapies	[19, 20]
	Specific gene examples	CDKN2A gene hypermethylation deactivates p16 and p15, disrupting cell cycle control by allowing unchecked progression from G1 to S phase, leading to increased cellular proliferation and tumor growth. **Key genes/molecules**: CDKN2A **Cancer types affected**: Breast, lung, colorectal, prostate, and others	Gene therapy, drugs restoring normal methylation patterns	[19, 20]
**Therapeutic strategies**	Nucleoside analogs	These analogs incorporate into DNA during replication, inhibiting DNMTs and leading to demethylation and potential reactivation of silenced tumor‐suppressor genes. **Key molecules**: Azacitidine, Decitabine **Cancer types**: Broad application	Used as frontline therapy or in combination with other epigenetic drugs	[23, 24]
	HDAC inhibitors	HDAC inhibitors prevent the deacetylation of histone proteins, resulting in a less compact chromatin structure and increased gene expression. This can counteract the silencing effects of hypermethylation on tumor‐suppressor genes. **Key molecules**: Vorinostat, Romidepsin **Cancer types**: Lymphomas, solid tumors	Used alone or in combination with DNMT inhibitors to enhance gene re‐expression	[23, 24]
	Combined epigenetic therapy	Combining DNMT and HDAC inhibitors with other targeted therapies like EZH2 inhibitors can synergistically restore normal gene function and inhibit tumor growth by reactivating tumor‐suppressor genes and modulating signaling pathways. **Key molecules**: Combination therapies **Cancer types**: Advanced and resistant cancers	Clinical trials exploring efficacy and safety in various cancer types	[23, 24]

### 2.4 Genomic Instability and Cancer

Global hypomethylation, another widespread phenomenon in cancer, contributes significantly to genomic instability. This reduction in methylation levels is particularly prevalent in repetitive DNA regions and among transposable elements, which, when demethylated, can become reactivated. Such activation leads to increased genomic instability due to the potential for these elements to insert themselves into critical genomic regions, disrupting gene function and regulatory regions. Furthermore, global hypomethylation can lead to chromosomal fragility, loss of imprinting, and activation of oncogenes, all of which are conducive to cancer progression and metastasis. This form of methylation dysregulation is a common feature in cancers such as colorectal, liver, and lung cancers, where it contributes to a more aggressive disease phenotype and poorer prognosis [25, 26, 27].

### 2.5 Impact of Hypermethylation and Hypomethylation in Critical Cellular Pathways

The hypermethylation of promoters of genes involved in essential cellular pathways—like cell cycle regulation, DNA repair, apoptosis, and cell adhesion—represents a common strategy by which cancers evade normal cellular controls. For instance, the hypermethylation of CHFR, a gene involved in cell cycle checkpoint regulation, allows cancer cells to bypass normal checkpoints in response to DNA damage, leading to further genetic aberrations. Similarly, the silencing of the DNA repair gene MGMT through methylation impedes the cell's ability to repair alkylated DNA, a common form of damage caused by both environmental factors and chemotherapy, thus increasing the mutation rate and contributing to chemoresistance. The inactivation of genes such as DAPK and Caspase 8, which are crucial for apoptosis, permits cancer cells to avoid programmed cell death, a key feature of cancer's resistance to therapies. Additionally, the methylation and consequent silencing of cadherin genes, which are vital for cell‐to‐cell adhesion, facilitate cancer metastasis by enabling cancerous cells to detach and spread to distant sites [25, 28].

Global hypomethylation refers to the widespread loss of methyl groups from cytosines in the genome, often seen in the repetitive DNA elements such as long interspersed nuclear elements (LINEs) and Alu repeats. This process contrasts with gene‐specific hypermethylation and is associated with genomic instability, reactivation of transposable elements, and altered gene expression [29]. For example, LINE‐1 elements are a type of repetitive DNA that constitute a significant portion of the human genome. They are normally heavily methylated to suppress their transposable activity. Hypomethylation of LINE‐1 elements is a common feature in various cancers, including colorectal, breast, liver, and lung cancers that can lead to increased transcriptional activity of these elements, contributing to genomic instability [9, 29]. Alu elements are short interspersed nuclear elements (SINEs) that are also highly repetitive and methylated under normal conditions to prevent their mobilization. Similar to LINE‐1, hypomethylation of Alu repeats is frequently observed in various malignancies [29] (Table 4).

**TABLE 4:** Types of DNA methylation, their functions, associated cancer, and application in liquid biopsy.

DNA hypermethylation marker		
**Epigenetic marker methylation**	**Function in cell**	**References**
CDKN2A/p16 and CDKN2/p15	Cell cycle regulation (regulates the passage from G1 into S phase by inhibiting pRb activation by phosphorylation) **Associated cancer**: Colorectal, lung, and pancreatic cancer. **Application**: Early diagnosis and prognosis	[9, 18, 19, 20]
RASSF1A and NORE1A/RASSF5	RAS signaling, regulation cell cycle progression, apoptosis, and microtubule stability **Associated cancer**: Lung, breast, genitourinary, prostate and colon cancers. **Application**: Early cancer detection and monitoring, tumorigenesis mechanisms	[9, 11, 25, 30]
APC	Regulator of the Wnt signaling pathway **Associated cancer**: Breast, prostate, lung, colorectal, gastrointestinal cancer **Application**: Early diagnosis and monitoring of treatment response	[9, 30, 31, 32]
MGMT	DNA repair, remove alkyl groups from guanine bases, protect from alkylating agents **Associated cancer**: Glioblastomas, colorectal, lung, lymphoma **Application**: Predict treatment response	[3, 9, 10, 25, 30, 33]
GSTP1	Detoxification of xenobiotics and carcinogens **Associated cancer**: Prostate, breast, kidney, genitourinary cancer **Application**: Distinguishing malignant from benign conditions	[3, 9, 34, 35]
SEPT9	Cell cycle control **Associated cancer**: Colorectal cancer **Application**: Early detection	[33]
SOX17	Downregulates the Wnt signaling pathway **Associated cancer**: Colorectal, lung, breast and hepatocellular carcinoma **Application**: Diagnostic and prognostic biomarker	[36]
HOXA9 and HOXA7	Involved in embryonic development and differentiation **Associated cancer**: Acute myeloid leukemia **Application**: Monitor disease progression and response to therapy	[36]
TIMP3	Inhibits metalloproteinases involved in extracellular matrix remodeling **Associated cancer**: Gastric, colorectal, glioblastoma, and renal cancer **Application**: Tumor invasion and metastasis predict	[37, 38, 39]

Epigenetic marker hypomethylation	Function in cell	References
LINE‐1 (long interspersed nuclear element‐1)	Maintains genomic stability; hypomethylation leads to genomic instability **Associated cancers**: Colorectal, breast, lung, gastric, hepatocellular carcinoma **Application**: Prognostic marker for tumor progression and poor survival	[40, 41]
Alu repeats	Regulates chromatin structure; hypomethylation causes genomic instability **Associated cancers**: Lung, bladder, colorectal, head and neck cancers **Application**: Indicator of advanced tumor stages and progression	[42, 43]
SAT2 (satellite 2)	Maintains chromosomal integrity; hypomethylation leads to chromosomal instability **Associated cancers**: Breast, ovarian, colorectal cancers **Application**: Marker for metastasis and chromosomal instability	[44, 45]
Global 5‐methylcytosine (5mC) content reduction	Reflects global DNA methylation status; hypomethylation leads to oncogene activation **Associated cancers**: Pancreatic, prostate, liver cancers **Application**: Diagnostic and prognostic biomarker for aggressive cancers	[46, 47]
MYC (oncogene) and MAGEA1 (melanoma antigen gene A1)	Oncogene activation due to promoter hypomethylation **Associated cancers**: Melanoma, lung cancer, hematological malignancies **Application**: Monitoring tumorigenesis and cancer progression	[48, 49]
IGF2 (insulin‐like growth factor 2)	Growth regulation; loss of imprinting leads to overexpression **Associated cancers**: Colorectal, liver, prostate cancers **Application**: Early detection marker, linked to tumor proliferation	[50, 51]
Pericentromeric satellite DNA	Ensures chromosomal stability; hypomethylation causes structural abnormalities **Associated cancers**: Breast, prostate cancers **Application**: Marker for chromosomal instability in advanced cancer stages	[52, 53]
Cancer‐testis antigen genes (e.g., NY‐ESO‐1, SSX)	Immune regulation; hypomethylation leads to aberrant expression in tumors **Associated cancers**: Lung, bladder, melanoma **Application**: Target for immunotherapy and cancer detection	[54, 55]

## 3 Histone Modification Epigenetic Markers in Liquid Biopsy

**FIGURE 3:**
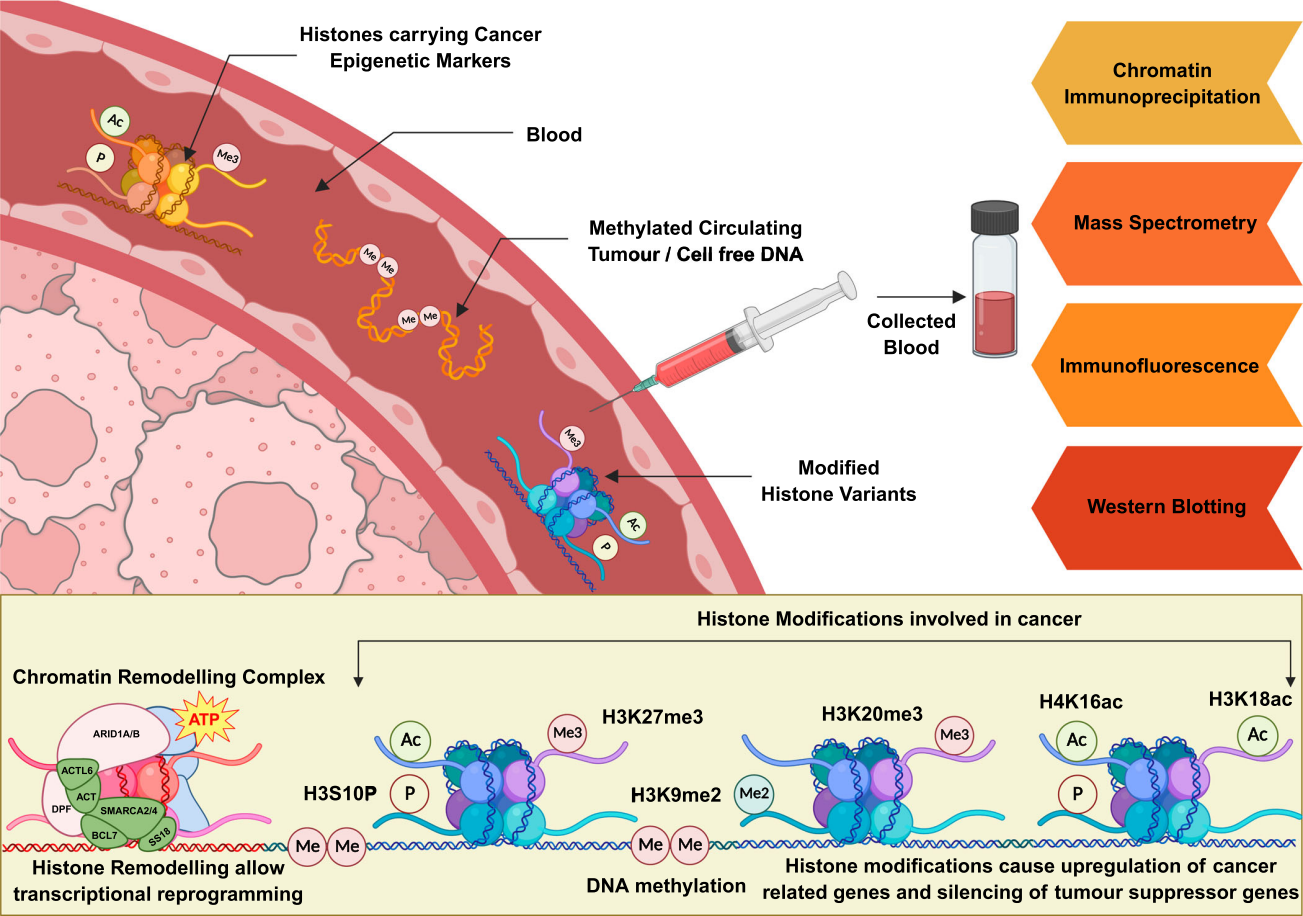
Histone modifications and chromatin remodeling involved in transcriptional reprogramming in cancer: This figure illustrates key epigenetic alterations—including histone acetylation, methylation, and chromatin remodeling—that contribute to dysregulated gene expression in cancer. These modified chromatin segments can be detected from blood (and other body fluids) by various techniques such as chromatin immunoprecipitation (ChIP), mass spectrometry, immunofluorescence assays, and Western blotting for diagnosis of cancer (created with BioRender.com).

The exploration of post‐translational modifications (PTMs) of histone proteins (PTHMs) in cancer research has provided profound insights into the molecular mechanisms governing chromatin dynamics and gene regulation [56]. These modifications, which include acetylation, methylation, phosphorylation, ubiquitination, and more, affect the interaction between histones and DNA, thereby influencing the chromatin's structure and the accessibility of transcriptional machinery to gene sequences (Figure 3). This epigenetic regulation is crucial for normal development and cellular homeostasis but can be perturbed in various diseases, including cancer.

**Diagnostic and Prognostic Applications**: Certain histone modifications are highly indicative of particular cancer types or states. For example, the acetylation of histone H3 at lysine 18 (H3K18ac) is linked to poor prognosis in several cancers, serving as a biomarker for aggressive disease. This specificity can aid in not only detecting cancer but also in predicting its behavior and potential response to treatments [57].

**Monitoring Treatment Response**: As patients undergo treatment, changes in the patterns of histone modifications can provide real‐time feedback on the effectiveness of the therapy. This is especially valuable for assessing responses to epigenetic drugs, such as HDAC inhibitors, which directly target these modifications. Sequential liquid biopsies can thus guide adjustments in treatment plans, optimizing therapeutic outcomes [57].

### 3.1 Oncogene Activation Through Histone Acetylation

The epigenetic modification of histone acetylation functions as a crucial process which determines how genes become expressed. The enzymes known as histone acetyltransferases (HATs) use acetyl groups to modify lysine residues located on histone tails. Acetylation of these groups creates neutralization effects on histone positive charges thus reducing their attraction to DNA's negative backbone. The decreased charge interaction creates an open chromatin structure that allows transcriptional machinery to access DNA and activates gene transcription. Histone acetylation functions as a key factor for activating oncogenes including MYC in cellular contexts [58]. The MYC gene produces a transcription factor which controls numerous genes that influence cell growth together with proliferation and metabolism and apoptosis. The MYC gene maintains strict regulatory control during normal body functions to keep cells in homeostasis. The proper balance between histone acetylation patterns gets disrupted when aberrant events occur which results in MYC overexpression and subsequent oncogenic transformation. Studies have shown MYC functions as a recruiter of HATs toward gene promoters which results in specific regions of histone hyperacetylation. The recruitment process strengthens MYC target gene transcriptional activation and involves genes that control cell cycle progression and cellular proliferation. The *Molecular and Cellular Biology* study revealed that MYC directs mitogen‐dependent histone H4 acetylation at particular target locations which requires this modification for gene activation [59].

The pathogenesis of different cancers includes MYC overexpression which results from increased histone acetylation. The MYC gene expression becomes excessively high in Burkitt's lymphoma patients through chromosomal translocations that connect it to powerful immunoglobulin promoters. The activity of MYC protein shows dysregulation in solid breast and colon tumors which leads to worse clinical outcomes and tumor progression. A *Journal of Hematology & Oncology* review demonstrates how MYC functions in cancer development while establishing its potential value as a therapeutic target [60].

The comprehension of MYC activation mechanisms through histone acetylation helps develop effective therapeutic methods. The acetylation process represents an attractive strategy to control MYC activity in cancer cells. Histone deacetylase inhibitors (HDACis) operate as compounds which stop histone deacetylase (HDAC) enzymes from removing acetyl groups from histones thus creating an active chromatin state that remains hyperacetylated. Research indicates that HDACis affect MYC activity by multiple mechanisms. Research in *Clinical Cancer Research* showed that HDACis cause MYC protein to become acetylated which leads to reduced MYC expression and apoptosis pathway activation in acute myeloid leukemia cells [58, 61].

The relationship between MYC and histone acetylation is indeed bidirectional. MYC not only recruit's HATs to promote histone acetylation but is also subject to acetylation itself, which can influence its stability and transcriptional activity.

Research has shown that MYC associates with the TIP60 HAT complex and recruits it to chromatin, leading to the acetylation of histones H3 and H4 at specific target genes. This recruitment facilitates transcriptional activation by creating a more open chromatin structure [62].

Additionally, MYC is acetylated by HATs such as GCN5 and TIP60. This acetylation enhances MYC's protein stability by inhibiting its ubiquitination and subsequent degradation. Consequently, acetylation modulates MYC's transcriptional activity by increasing its persistence within the cell [63].

### 3.2 Silencing of Tumor‐Suppressor Genes Through Histone Methylation

EZH2 functions as the catalytic subunit of PRC2 to serve as a histone‐lysine N‐methyltransferase enzyme. EZH2 performs the essential role of H3K27me3 trimethylation to histone H3 lysine 27 which results in chromatin compaction and transcriptional repression. The epigenetic mechanism controls development and cell identity maintenance through its vital role in gene expression regulation. The development of various cancers seems to be influenced by EZH2 dysregulation because it leads to tumor‐suppressor gene silencing [58].

EZH2‐mediated H3K27me3 functions normally to silence differentiation‐promoting genes which maintains stem cell pluripotency while controlling developmental processes. The developmental genes that embryonic stem cells need to silence for pluripotency maintenance operate under PRC2 regulation through EZH2. The gene RUNX3 represents one of the developmental genes that EZH2 controls through its regulatory functions [64].

Mutations or improper expression of EZH2 protein results in abnormal gene silencing processes. EZH2 overexpression occurs frequently in different types of cancer including melanoma while its activity leads to gene silencing through DNA methylation of tumor‐suppressor elements. The combination of EZH2 with DNA methylation causes the silencing of important suppressor genes which drives melanoma tumor development [58, 65].

The elevated activity of EZH2 enhances H3K27 methylation which leads to the silencing of tumor‐suppressor genes. The catalytic properties of EZH2 play a crucial role in cancer development and progression thus making it an attractive therapeutic target [66].

The cancer‐suppressing genes become silenced through EZH2‐mediated histone methylation events across different cancer types. The disease process of cholangiocarcinoma leads to cancer initiation through tumor‐suppressor gene silencing and chromosomal instability caused by aberrant histone methylation [67].

Research has turned EZH2 into a promising target because it plays a vital role in gene silencing processes. The goal of EZH2 inhibitor drugs is to break tumor‐suppressor gene silencing which leads to cancer cell death and prevents their proliferation. Tazemetostat acts as an EZH2 inhibitor by blocking the EZH2 methyltransferase activity which leads to gene reactivation and tumor growth suppression in specific cancers [68].

EZH2‐mediated histone methylation acts as a fundamental mechanism to silence tumor‐suppressor genes epigenetically. The normal development and cellular function depend on EZH2 but its dysregulation leads to cancer formation through gene repression which blocks tumor inhibition. The therapeutic potential of EZH2 pathway inhibition together with its associated mechanisms shows promise as a method to restore tumor‐suppressor genes and fight cancer development.

### 3.3 Circulating Nucleosomes and Histone Modifications: Emerging Biomarkers in Cancer Diagnosis and Therapy

Circulating nucleosomes represent the essential chromatin units consisting of DNA wrapped around histone proteins which demonstrate potential as diagnostic biomarkers in cancer testing. The bloodstream receives nucleosomes from cells that die through apoptosis or necrosis during cellular turnover especially in tumor microenvironments. The circulating nucleosomes maintain unique histone PTMs that reveal information about the epigenetic nature of tumors [57].

Medical research shows that cancer patients display higher nucleosome concentrations in their plasma and serum than healthy subjects. The diagnostic value of nucleosomes for cancer detection is restricted by their ability to increase in benign conditions as well as malignant conditions. The distinctive modifications found on nucleosomes help researchers gain meaningful information [54]. The presence of tumor cells along with their biological behavior becomes evident through specific PTMs that activate or repress genes. The examination of these modifications supports the separation of malignant from nonmalignant conditions [69].

Research investigations demonstrate that blood‐based biomarkers for early cancer detection can be achieved through the identification of particular histone modifications found on circulating nucleosomes. Scientists have discovered that detecting modifications in histone patterns on circulating nucleosomes creates a strong diagnostic tool which helps identify cancer before it advances [70].

The examination of circulating nucleosomes has been conducted for particular cancer types. Scientists have discovered that specific histone modifications on circulating nucleosomes demonstrate potential for pancreatic cancer diagnosis. Nucleosome‐based assays demonstrate great potential for pancreatic cancer diagnosis and monitoring according to this research approach [57, 71].

The diagnostic value of circulating nucleosomes reaches further than initial detection. The evaluation of nucleosome levels together with their modifications enables the assessment of tumor progression as well as treatment responses and disease evolution. A blood test which combined circulating nucleosomes targeting different epigenetic modifications with carcinoembryonic antigen (CEA) enabled the detection of lung cancer cases in nonsmokers according to research findings [72].

The analysis of circulating nucleosomes presents an invasive‐free method for researchers to obtain tumor‐specific epigenetic data. Clinical professionals use histone modification analysis of circulating nucleosomes to obtain crucial cancer information which helps them detect cancers early and monitor disease progression and select individualized treatments. Research progress indicates that circulating nucleosome analysis will soon become a valuable tool for cancer diagnosis and treatment outcome improvement in clinical settings.

Technological advancements in areas like next‐generation sequencing (NGS) and enhanced mass spectrometry are rapidly improving our ability to detect and quantify histone modifications. These improvements will likely usher in a new era of precision oncology, where histone modification profiles could routinely inform clinical decisions, from diagnosis through to prognostication and therapeutic intervention. Moreover, as our understanding of the complex interactions among various PTHMs deepens, new targets for drug development will emerge, potentially leading to novel treatments that specifically modify the epigenetic landscape of cancer cells. The study of PTHMs in cancer provides critical insights into the regulatory mechanisms of gene expression and offers promising avenues for the development of novel diagnostic and therapeutic strategies. Liquid biopsy stands out as a particularly impactful application, promising to transform cancer management through minimally invasive, repeatable, and highly informative assessments of the disease (Table 5).

**TABLE 5:** Post‐translational modifications of histone proteins (PTHMs) in the context of cancer research, diagnostics, and therapeutics.

Category		Description	Cancer implications	References
**Mechanisms of PTHMs**	Oncogene activation	Histone acetylation, facilitated by histone acetyltransferases (HATs), promotes transcriptional activation by loosening chromatin structure around oncogene loci. This process enhances gene expression related to cell growth and division. **Key modifications/genes**: Acetylation (e.g., H3K9ac, H3K27ac)	Critical in the upregulation of oncogenes such as MYC and BCL‐2, driving malignant transformation and cancer cell survival in numerous cancer types, including breast cancer and lymphomas.	[57]
		The MLL1 core complex, including ASH2L, WDR5, and RBBP5, mark H3K4 on histones at gene promoters **Key modifications/genes**: Methylation (e.g., H3K4me3)	Activates oncogenes and promoting transcriptional changes that drive cancer cell growth.	
	Tumor‐suppressor gene silencing	Histone methylation, often mediated by histone methyltransferases (HMTs) like EZH2, results in chromatin compaction, silencing tumor‐suppressor genes by adding methyl groups to specific lysine residues on histones. **Key modifications/genes**: Methylation (e.g., H3K27me3, H3K9me3)	Commonly leads to the repression of genes that control cell cycle arrest and apoptosis, promoting tumor progression in cancers such as prostate cancer and glioblastoma.	[73, 74]
	Chromatin remodeling and stability	Phosphorylation and ubiquitination of histones can alter the structural dynamics of chromatin, affecting DNA repair mechanisms and chromosomal stability during cell division and in response to DNA damage. **Key modifications/genes**: Phosphorylation (e.g., H3S10ph), Ubiquitination	These modifications are pivotal in the cellular response to DNA damage and stress, influencing pathways involved in cancer progression and resistance to chemotherapy, notably in ovarian and colorectal cancers.	[2]
**Diagnostic applications in liquid biopsy**	Epigenetic biomarkers for detection	Circulating nucleosomes containing specific histone modifications can be detected in blood samples, providing insights into the type and state of cancer based on the epigenetic landscape reflective of the primary tumor's chromatin organization. **Key modifications/genes**: Various specific PTHMs	Enables the detection and characterization of cancers at an early stage, improving the accuracy of noninvasive diagnostics and potentially aiding in the screening for high‐risk populations.	[3, 7, 8]
	Prognostic significance	Certain histone modifications correlate with cancer prognosis, where specific patterns such as increased methylation or acetylation at defined sites can indicate tumor aggressiveness, likelihood of metastasis, and overall survival rates. **Key modifications/genes**: H3K18ac, H4K20me3	Useful for stratifying patients based on risk, guiding treatment decisions, and predicting outcomes in cancers like prostate and lung cancer. These markers provide a window into the tumor's behavior and potential response to therapies.	[57]
	Monitoring therapeutic response	Sequential liquid biopsies showing changes in histone modification patterns can reflect the tumor's response to treatment, especially targeted epigenetic therapies. This dynamic monitoring helps in adjusting treatment strategies based on real‐time data. **Key modifications/genes**: Various specific PTHMs	Critical for evaluating the effectiveness of epigenetic drugs and other treatments, allowing for personalized adjustments in therapy to optimize clinical outcomes, particularly in the treatment of hematologic malignancies and solid tumors.	[1, 7]
**Therapeutic implications and future prospects**	Targeted epigenetic therapy	Drugs targeting specific enzymes involved in histone modifications, such as HDAC inhibitors, HMT inhibitors, and BET inhibitors, are being developed and used to modify the epigenetic landscape of cancer cells, aiming to reverse malignant phenotypes. **Key modifications/genes**: HDAC inhibitors, EZH2 inhibitors	These therapies offer new avenues for treating cancers that are resistant to conventional therapies by specifically altering the epigenetic regulation mechanisms, showing promise particularly in lymphomas and advanced solid tumors.	[49]
	Advances in detection technologies	Ongoing improvements in mass spectrometry and next‐generation sequencing technologies are enhancing the sensitivity and specificity of detecting histone modifications, allowing for more detailed profiling and understanding of cancer epigenetics.	These technological advancements will likely lead to the routine clinical integration of histone modification analysis, offering more precise diagnostics and tailored treatment plans, further pushing the boundaries of personalized medicine in oncology.	[3, 7, 8]

In eukaryotic cells, where histone acetylation and methylation are ubiquitous chromatin marks and important regulators of gene expression, a variety of PTHMs have been discovered. These have mostly been linked to the onset and spread of cancer [75]. Since acetylation alters the net positive charge of histone proteins and makes DNA sequence information accessible, it is commonly linked to transcriptional activation of genes. HATs and HDACs maintain a dynamic equilibrium that determines the acetylation status of the histones. HDACs reverse histone acetylation and impact the expression of several cancer‐critical genes by eliminating acetyl groups. Numerous cancers have been connected to aberrant expression of HDACs [76]. They are thought to be implicated in many stages of cancer, and in the majority of cases, advanced illness and unfavorable patient outcomes are linked to high HDAC levels. HDACs are therefore a pertinent target for cancer treatments [7]. Histone lysine methyltransferases (HKMTs) and protein arginine methyltransferases (PRMTs) add one to three methyl groups to the side chains of lysine or arginine residues, respectively, to methylate histones [77]. HKMTs and PRMTs add one to three methyl groups to the side chains of lysine or arginine residues, respectively, to methylate histones. The number of methyl groups and where they are located within the histone tail determines the functional effects of methylation [73].

### 3.4 Histone Modifications as Cancer Biomarkers: Current Obstacles and Emerging Solutions

The detection of histone modifications in blood and urine samples remains a complex task. The low concentration of these molecules makes direct measurement challenging so researchers analyze cfDNA or nucleosome‐associated DNA fragments instead to detect modifications. The regulation of gene expression depends on histone modifications including methylation and acetylation which show promise as cancer biomarkers. The identification of modifications between normal and cancer cells in biofluids proves to be a complex task [73]. Table 6 summarizes the key differences between normal and cancer‐associated histone modification patterns.

**TABLE 6:** Impact of noncoding RNAs in critical cellular pathways.

ncRNA type		Description		References
		Mechanisms and roles in cancer	Potential in therapy and liquid biopsy	
**miRNAs**	miR‐21	Acts as an oncomiR by inhibiting tumor‐suppressor genes such as PTEN and PDCD4, activating the PI3K/AKT pathway for cell survival and proliferation. **Cancer association**: Breast, lung, colorectal, glioblastoma	Target for therapies aimed at reactivating tumor‐suppressor pathways; biomarker for tumor presence and progression in liquid biopsies.	[9, 100]
	miR‐34a	Induced by p53 and targets genes involved in cell cycle progression like CDK4/6 and E2F3, promoting apoptosis. **Cancer association**: Colorectal, lung, pancreatic	Used to enhance p53 pathways in treatment; levels in blood can indicate response to therapies targeting p53‐related pathways.	[55, 146]
	miR‐200 family	Regulates EMT by targeting TGF‐β signaling components ZEB1 and ZEB2, reducing metastatic capabilities. **Cancer association**: Ovarian, breast, prostate, colorectal	Potential targets to inhibit EMT in therapeutic strategies; markers for metastatic potential and EMT status in liquid biopsies.	[102]
	miR‐15/16	Functions as tumor suppressors by regulating cell cycle and apoptosis, commonly downregulated in specific cancers. **Cancer association**: CLL, prostate	Restoration in therapies could inhibit cancer progression; useful as prognostic markers in liquid biopsy.	[97, 98]
	miR‐221/222	Act as oncomiRs by targeting tumor suppressors p27 and p57, promoting cell proliferation. **Cancer association**: Breast, hepatocellular, prostate, glioblastoma	Candidates for targeted miRNA silencing therapies; elevated levels may serve as diagnostic markers.	[101]
	miR‐155	Upregulated in non–small‐cell lung cancer (NSCLC), associated with metastasis. **Cancer association**: Lung cancer, pancreatic cancer	Sensitive to detect NSCLC in liquid biopsy, also linked to poor prognosis.	[147, 148]
	miR‐141	Upregulated in colorectal cancer, associated with diagnosis. **Cancer association**: Colorectal cancer	Sensitive to liquid biopsy detection, used for noninvasive screening and monitoring.	[148]
	miR‐141	Upregulated, linked to tumor progression in prostate cancer. **Cancer association**: Prostate cancer	Detected in plasma as a marker for prostate cancer diagnosis and prognosis.	[149]
**lncRNAs**	HOTAIR	Recruits PRC2 to silence genes at the HOXD locus, facilitating metastasis and progression. **Cancer association**: Breast cancer, others	Inhibiting HOTAIR could reverse gene silencing; its levels in fluids can predict metastatic risk.	[107, 108]
	MALAT1	Associated with metastasis by influencing gene expression linked to motility and invasiveness. **Cancer association**: Lung cancer, others	Targeting MALAT1 may reduce metastatic behavior; serves as a marker for invasive cancer types.	[107]
	GAS5	Acts in growth arrest and apoptosis, often downregulated in cancer, influencing cell survival. **Cancer association**: Prostate cancer, others	Potential therapeutic agent to promote apoptosis in cancer cells; diagnostic marker for growth arrest status.	[150]
	HULC	Overexpressed in non–small‐cell lung cancer, linked to tumor progression. **Cancer association**: Lung cancer	Can be detected in liquid biopsy to assess lung cancer progression and metastasis.	[151]
	CCAT1	Upregulated, plays a role in tumorigenesis and cell migration. **Cancer association**: Colorectal cancer	Liquid biopsy markers for noninvasive diagnosis and monitoring of tumorigenesis.	[108]
	PCAT1	Upregulated in prostate cancer, correlates with tumor progression. **Cancer association**: Prostate cancer	Potential for liquid biopsy‐based diagnostic tool and therapy monitoring.	[152]
	UCA1	Upregulated, associated with poor prognosis and metastasis. **Cancer association**: Pancreatic cancer	Potential liquid biopsy marker for early detection and metastasis monitoring in pancreatic cancer.	[153]

#### 3.4.1 Comparison of Histone Modification Patterns in Normal and Cancer Cells

In normal cells, histone acetylation is balanced and gene‐specific, ensuring controlled gene expression. In contrast, cancer cells often exhibit altered acetylation patterns, which lead to dysregulated gene expression [78]. Similarly, histone methylation in normal cells is tightly regulated to maintain proper gene function, whereas in cancer cells, this regulation becomes disrupted. For instance, the loss of H3K27me3 due to mutations in EZH2 can result in aberrant gene activation [79]. Regarding global chromatin patterns, normal cells maintain consistency across different cell types, while cancer cells display heterogeneous, tumor‐specific epigenetic landscapes [80].

#### 3.4.2 Challenges in Distinguishing Histone Markers

One major challenge in utilizing histone modifications for cancer detection is the heterogeneity of these modifications. Histones undergo diverse PTMs, such as methylation, acetylation, and phosphorylation, across different cell and tissue types, making it difficult to identify generic cancer‐specific patterns. Cancer‐associated modifications like H3K9me3 and H4K20me3 are also present in healthy cells, limiting their specificity as reliable biomarkers. The wide range of modifications complicates efforts to distinguish cancer cells from normal cells, highlighting the need for more precise epigenetic markers [81]. Another challenge lies in the low abundance of histone‐modified proteins in circulating fluids such as blood and urine. Although sensitive detection techniques have recently improved identification success, the extremely low levels of these proteins pose significant obstacles. For instance, a study showed that nickel exposure could alter global histone modification patterns in peripheral blood mononuclear cells, demonstrating that blood‐based measurements are feasible. Similarly, researchers have explored DNA methylation and histone modification profiles in urine samples to advance bladder cancer detection through noninvasive urinary testing [82, 83]. Technical limitations further complicate the detection of histone modifications. The minimal concentrations of circulating histone modifications, combined with the tissue‐intensive requirements of chromatin immunoprecipitation sequencing (ChIP‐seq), restrict its application for liquid biopsy. The scarcity of cell‐free nucleosomes in fluids like blood and urine demands highly sensitive and innovative detection methods. Recent studies are investigating how histone modifications correlate with cfDNA fragmentation patterns, offering potential new approaches to noninvasively analyze histone modifications via cfDNA profiling [84].

#### 3.4.3 Potential Solutions and Future Directions

To overcome these challenges, advanced analytical techniques and a deeper understanding of cancer‐specific histone modification patterns are required.

**Advanced Detection Methods**: The identification of histone modifications in cancer samples remains difficult because of copy number variations together with GC‐content bias. The analysis of cancer genome ChIP‐seq data requires tools like HMCan to handle these problems. The tool HMCan addresses GC‐content and copy number variations biases which makes histone modification detection more precise [85].

**Comprehensive Histone Modification Profiling**: The development of a full quantitative atlas of histone modification signatures throughout distinct cancer types remains vital for cancer‐specific signature identification. A reference database built for this purpose would help identify suspicious patterns that indicate malignant development while improving diagnostic precision and therapy selection. A proteomic atlas of cancer lines based on histone modification patterns was developed by Leroy et al. which now serves researchers who study cancer epigenetics [86].

**Integration With Other Biomarkers**: The combination of histone modification analysis with DNA methylation patterns and ctDNA biomarkers improves cancer detection accuracy. A complete analysis through this method reveals detailed information about cancer‐related epigenetic changes. The cancer clinic now uses ctDNA methylation as a promising molecular marker for noninvasive testing [87]. The combination of histone modification analysis with ctDNA profiling enables researchers to study tumor‐specific epigenetic changes which results in better diagnostic accuracy [88].

Advancements in these techniques bring us closer to utilizing histone modifications as reliable biomarkers for cancer detection and monitoring. Ongoing research focuses on improving methods for extracting histone‐modified DNA and developing more sensitive detection instruments. Combining histone modification data with other biomarkers, such as DNA methylation and miRNAs, enhances diagnostic precision. As technology advances, histone modification profiling is poised to become a central tool in noninvasive cancer detection, early diagnosis, and personalized treatment strategies.

## 4 Noncoding RNAs Epigenetic Markers in Liquid Biopsy

**FIGURE 4:**
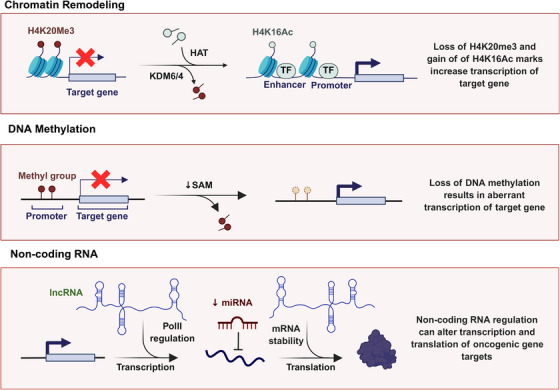
Coordinated epigenetic mechanisms in cancer progression: chromatin remodeling, DNA methylation, and noncoding RNAs collectively disrupt gene expression programs to drive cellular transformation and tumor development, highlighting their roles in silencing tumor suppressors, activating oncogenes, and promoting malignant phenotypes (created with BioRender.com).

ncRNAs, which include (miRNAs, lncRNAs, play crucial regulatory roles in gene expression without coding for proteins (Figure 4). These molecules are remarkably stable in bodily fluids like blood and urine, making them suitable for noninvasive testing [3, 89]. miRNAs are small, ncRNA molecules that regulate gene expression by targeting messenger RNA (mRNA) for degradation or translational repression [31, 90]. They are small, ncRNA molecules, typically 20–24 nucleotides long, that play crucial roles in gene regulation by binding to complementary sequences on mRNA transcripts, leading to their degradation or inhibition of translation [91]. Cells release miRNAs in conjunction with RNA‐binding proteins or bundled inside exosomes, which shield the cell‐free miRNAs from RNase action [2, 92]. In the context of cancer, miRNAs can function as oncogenes (oncomiRs) or tumor suppressors, influencing various cellular pathways that drive tumorigenesis [93, 94].

### 4.1 miRNAs as Epigenetic Markers

One of the most studied oncomiRs, miR‐21 targets and suppresses the expression of tumor‐suppressor genes such as PTEN and PDCD4 that activate the PI3K/AKT pathway promoting cell survival, proliferation, and growth [95]. This leads to enhanced cell proliferation and reduced apoptosis, promoting tumor growth in cancers such as breast, lung, prostate, colorectal cancer, and glioblastoma [9]. Evaluation continuously shown elevated levels of circulating miR‐141, miR‐375, miR‐221, miR‐93, and miR‐21 in the plasma of prostate cancer patients [96]. miR‐15 and 16 functions as a tumor suppressor, regulating cell cycle and apoptosis. Reduced levels of miR‐16 are seen in cancers such as chronic lymphocytic leukemia (CLL) and prostate cancer [97, 98]. miRNAs such as miR‐29 and miR‐34 target components of the Wnt/β‐catenine signaling pathway, which is often deregulated in cancers. By modulating this pathway, miRNAs influence cell proliferation, differentiation, and stem cell renewal. Induced by p53, miR‐34a targets genes involved in cell cycle progression, such as CDK4/6 and E2F3 [99]. miR‐21 is downregulated in various cancers, including colorectal, lung, and pancreatic cancers [100]. miR‐221 and miR‐222 are oncomiRs that target tumor suppressors like p27 and p57 [101, 102]. Overexpression of miR‐221/222 is seen in breast cancer, hepatocellular carcinoma (HCC), prostate cancer, and glioblastoma [9, 101]. Members of the miR‐200 family (miR‐200a, miR‐200b, miR‐200c) regulate the TGF‐β pathway, which plays a crucial role in epithelial‐to‐mesenchymal transition (EMT), a process that enhances metastatic potential. By targeting TGF‐β signaling components, ZEB1 and ZEB2, transcription factors that promote EMT, the miR‐200 family suppress the mesenchymal phenotype, reducing invasive and metastatic capabilities of cancer cells [95]. Loss of these miRNAs is linked to increased metastatic behavior in cancers such as ovarian, breast, prostate and colorectal cancer [9, 103].

### 4.2 lncRNAs as Epigenetic Markers in Liquid Biopsy

Liquid biopsy diagnostics and monitoring show great potential through the use of lncRNAs as important epigenetic markers. These RNA molecules measure more than 200 nucleotides in length without creating proteins and function to manage gene expression and sustaining cellular processes. LncRNAs modify epigenetic states through direct binding to chromatin and transcription factors and regulatory proteins which enables gene expression control of cancer promoting genes. LncRNA detection through liquid biopsy occurs through examination of blood and urine alongside saliva samples which present different expression patterns than healthy subjects. Biomarkers made from these compounds offer significant value for cancer screening and evaluation and therapy response tracking. This testing technique displays the molecular tumor composition of distant areas using blood testing which provides less invasive analysis than invasive biopsies do. Research on lncRNA demonstrates their capability to monitor tumor progressive events such as metastasis and therapeutic resistance and disease recurrence along with tracking dynamical tumor alterations. The field of lncRNA biology exploration keeps advancing to improve their performance in liquid biopsy epigenetic markers which creates new opportunities for individualized cancer medicine [104].

Scientists have discovered that long ncRNAs MALAT1 and HOTAIR demonstrate substantial value for liquid biopsy exams as cancer diagnostic and prognostic indicators across numerous cancer types. The Metastasis‐Associated Lung Adenocarcinoma Transcript 1 (MALAT1) controls gene expression and alternative splicing functions while its abnormal control has been associated with cancer development in lung cancer and breast cancer and colorectal cancer cases. The high levels of MALAT1 expression serve as an important biomarker for liquid biopsy because they signal poor outcomes and metastasis development during cancer detection and monitoring [94, 105, 106]. The well‐known lncRNA molecule HOTAIR (HOX Transcript Antisense RNA) determines gene silencing outcomes while aiding chromatin remodeling activities. Scientists have identified HOTAIR overexpression as a marker that indicates tumor invasion and metastasis together with reduced clinical effectiveness in breast cancer and liver cancer and gastric cancer (GC) cases [107, 108].

### 4.3 Exosomal miRNAs as Cancer Biomarker

Lipid‐bilayer nanovesicles (30–150 nm) secreted by most cells, known as exosomes, are becoming recognized as strong biomarkers in liquid biopsy [109, 110, 111]. They possess miRNAs, mRNAs, and other RNAs protected against degradation, and cell‐specific surface proteins which indicate where they came from [109, 112, 113]. Exosomal miRNAs feature as one of the most exciting tumor biomarkers among their loads because they are stable and tumor‐specific. Research indicates that exosomal miRNAs can identify early‐stage tumors with more specificity and consistency of cancer‐derived miRNAs from biological fluids such as blood, urine, and saliva [111, 113, 114, 115]. In addition to miRNAs, exosome surface proteins (e.g., CD63, CD81, tumor‐associated antigens) provide a phenotypic dimension to the diagnostics that enhances the sensitivity and facilitates a multiple‐plexed‐analysis [22, 112]. For instance, exosomal miRNAs such as miR‐200b‐3p, miR‐3124‐5p, and miR‐92b‐5p have been identified as diagnostic and prognostic markers in small‐cell lung cancer (SCLC), while miR‐21‐5p and miR‐155 are consistently elevated in non–small‐cell lung cancer (NSCLC) as well as constantly overexpressed in the exosomes of breast and pancreatic cancer patients and correlate with tumor progression [22, 116, 117, 118]. RNA sequencing identified miR‐223‐3p as significantly upregulated in neutrophil‐derived exosomes (Neu‐Exo) as biomarkers for GC [119]. Similarly the levels of exosomal miR‐1246 and miR‐1290 in pancreatic ductal adenocarcinoma (PDAC) patients are good predictors of chemotherapy response [120]. Combinations of exosomal miRNA signatures with ctDNA methylation signatures are being considered as innovative ways to enrich multianalyte cancer detection capabilities [121].

It was also found from that miR‐361‐3p and miR‐625 distinguish malignant and benign lesions in lung cancer and miR‐21 helps early diagnosis of the squamous cell carcinoma in lung [122, 123, 124]. In lung cancer patients compared to bronchoalveolar lavage fluid, exosomal miR‐122‐5p is upregulated and miR‐200b‐5p, miR‐378, miR‐502‐5p and miR‐629 are upregulated in lung adenocarcinoma in comparison with granuloma or healthy smokers [124, 125, 126]. miR‐21 and miR‐155 are used as diagnostic markers, whereas post‐re Exosomal leucine‐rich alpha‐2‐glycoprotein 1 (LRG1) is overexpressed in NSCLC, and the exosomal miR‐21/miR‐155 level is increased in a recurrent tumor [127, 128, 129, 130]. In cancer of the digestive system cancers, exosomal miR‐665 is related to HCC tumor size and stages, miR‐718 is under expressed in HCC, miR‐122, miR‐148a, and miR‐1246 are over expressed in HCC versus liver cirrhosis and exosomal miR‐21 is more sensitive than serum miR‐21 [131, 132, 133]. The let‐7 family members stimulate the metastasis in GC, and miR‐1225‐5p/miR‐320c would forecast peritoneal recurrence [134, 135]. The group of colorectal cancer presents highly expressed exosomal let‐7a, miR‐1246, and miR‐23a (sensitivity compared to CA19‐9/CEA in the stage I), whereas miR‐17‐92a cluster prospects recurrence [136, 137]. CA19‐9 is complemented by upregulated miR‐1246 and miR‐4644 in pancreatic cancer patients [138]. In genitourinary malignancies, notable examples of prostate cancer biomarkers are miR‐1246 associated with aggressiveness, miR‐1290/miR‐375 as biomarkers of prognosis in carcinoma of the prostate that is resistant to castration, and miR‐141 and metastasis [139, 140, 141]. In ovarian cancer, miR‐21 and miR‐200 family differentiate between benign and malignant tumors, whereas miR‐1290 can differentiate between high‐grade serous cases [142, 143]. miR‐375/miR‐1307 are related to the presence of lymph node metastasis [144]. Exosome in cervicovaginal lavage contains high concentration of miR‐21/miR‐146a in cervical cancer cells [145].

### 4.4 Impact of Noncoding RNAs in Critical Cellular Pathways

The dysregulation of lncRNAs and miRNAs in cancer provides a molecular basis for distinguishing cancer cells from normal cells. Various miRNAs and lncRNAs, detailing their functions, roles in cancer pathology, and therapeutic implications. This expanded format provides clearer insights into their specific contributions to cancer development and potential roles in treatment strategies.

### 4.5 Challenges in Detecting lncRNA and miRNA as Biomarkers in Liquid Biopsy

Liquid biopsy has achieved notable attention for biomarker usage of lncRNA and miRNA because of their ability to perform noninvasive disease diagnosis and monitoring. Different technical obstacles and biological barriers restrict their adoption in clinical settings. The application of lncRNAs and miRNAs in liquid biopsy encounters problems stemming from RNA stability issues alongside challenges with isolation and measurement methods and factors determined by biological variability and the absence of standardized procedures. These are the main obstacles in detecting lncRNAs and miRNAs from liquid biopsy applications.

#### 4.5.1 RNA Stability and Degradation

The use of miRNAs and lncRNAs in liquid biopsies faces the major challenge due to their fragile nature that leads to decomposition. Because of their short size and Argonaute protein complex miRNAs stand up well to degradation but the larger lncRNAs become easily destroyed by RNases found in blood as well as other bodily fluids [154]. Biomarker detection becomes inaccurate because of this instability that affects diagnostic precision. Research demonstrates that packaged miRNAs and lncRNAs inside exosomes retain better stability compared to their free versions whereas their extraction method requires advanced technical expertise [155]. RNA integrity gets compromised by inconsistent sample handling and delayed processing time and improper storage conditions which results in unreliable experimental outcomes.

#### 4.5.2 Low Abundance and Sensitivity of Detection Methods

It becomes challenging to detect disease‐associated lncRNAs and miRNAs since they exist at extremely low expression levels within bodily fluids [156]. Accurate expression measurement requires special detection methods like quantitative real‐time PCR (qRT‐PCR), digital droplet PCR (ddPCR), and NGS because these molecules exist at very low levels. However, these methods have limitations:

The analysis through qRT‐PCR and ddPCR becomes restricted because they need previously known target sequences to operate effectively as detection tools [157].

The RNA profiling capability of NGS establishes broad characterization yet its high cost associates with advanced bioinformatics solutions needed to analyze data [158].

The detection methods display high variability and background noise that diminishes result reproducibility [159].

#### 4.5.3 Variability in RNA Isolation and Extraction Methods

The success rate of RNAs extracted from liquid biopsies depends on both the specimen type (plasma, serum, urine, saliva) combined with the isolation kit selection along with extraction protocol utilization. The variance in extraction efficiency causes conflicting RNA yield and purity levels which negatively affects subsequent analytical tests [160].

The extraction methods for acquiring lncRNA molecules from biofluids become more challenging due to their cellular retention and exosome retention properties compared to miRNAs [161]. The isolation procedure of RNA becomes complicated due to blood sample contaminants that include heme and proteins which negatively affect RNA quality [162].

#### 4.5.4 Lack of Standardization and Reference Controls

Standard protocols for collecting and isolating RNA and normalizing data represent significant barriers which block the clinical implementation of lncRNAs and miRNAs as biomarkers. Variability in:

- Sample collection (plasma vs. serum)
- RNA isolation kits

The evaluation of two data normalization methods uses internal controls which include U6 and synthetic spike‐in RNAs.

The inconsistency in research practices causes significant effects on data findings consistency between different research groups [163]. Standardized approaches need to become essential because they guarantee reliable clinical usage of these biomarkers.

#### 4.5.5 Biological Variability and Disease‐Specific Expression

Multiple biological and environmental elements influence the expression patterns of miRNAs and lncRNAs as reported in research studies [164]. The pattern of expression varies between individuals as well as according to specific stages of disease and particular tissue types or genetic patterns. The wide range of biological characteristics in human samples hinders scientists in developing common biomarkers to detect diseases [165]. The measurement of certain miRNAs in circulation could become unreliable when their levels change because of physiological variations not linked to the diagnostic aim [166].

#### 4.5.6 Interference from Contaminating RNAs and Nonspecific Binding

The detection of miRNAs and lncRNAs in liquid biopsy faces difficulties because of the interference from background RNAs as well as nonspecific binding to other molecules. For instance: During sample collection the process of hemolysis causes cellular miRNAs to contaminate samples and this leads to false biomarker profiles [167]. The binding between lncRNAs and RNA‐binding proteins during analysis processes leads to difficulties regarding their quantification and interpretation [168].

## 5 Summary of Detection Assays for Epigenetic Markers in Liquid Biopsy

A potent noninvasive method for identifying epigenetic markers in cancer and other illnesses is liquid biopsy. The detection tests for epigenetic markers, including as DNA methylation, histone modifications, and ncRNAs, are summarized in tabular form below, along with current research demonstrating these developments (Table 7).

**TABLE 7:** Detection assays for epigenetic markers in liquid biopsy.

Detection assays for epigenetic markers		Key features	Applications in cancer diagnostics	References
Marker	Detection assay			
DNA methylation	Bisulfite sequencing	Converts unmethylated cytosines to uracil; detects methylated cytosines	Early detection of colorectal cancer	[169, 170, 171, 172]
	Methylation‐specific PCR (MSP)	Amplifies methylated DNA regions using specific primers	Detection of lung cancer	[170, 173]
	Digital droplet PCR (ddPCR)	Quantifies methylated DNA with high sensitivity and specificity	Monitoring breast cancer recurrence	[171, 174]
	CRISPR/dCas9‐DNMT3A/TET1	dCas9‐DNMT3A fusions can hypermethylate oncogene promoters (e.g., MYC), while dCas9‐TET1 reactivates tumor suppressors	Reactivating silenced tumor suppressors (CDKN2A, BRCA1) and silencing oncogenes (MYC, KRAS).	[175]
	Bisulfite‐free cyclic voltammetry analysis	Redox reactions of methylated cytosines at electrode surface with zeptomolar sensitivity	Pan‐cancer (e.g., colorectal, lung)	[176, 177, 178]
	SERS (surface‐enhanced Raman spectroscopy)	The MBD1 protein conjugated with a DTNB Raman reporter binds methylated DNA, and plasmonic coupling between nanoparticles subsequently amplifies the DTNB signal for detection	Colorectal, glioblastoma profiling	[179, 180, 181]
Histone modifications	Chromatin immunoprecipitation (ChIP)	Identifies histone modifications using antibodies against specific PTMs	Profiling H3K27me3 in ovarian cancer	[182, 183]
	Mass spectrometry	Quantifies histone PTMs with high resolution	Detection of prostate cancer in urine	[182, 183]
	ELISA‐based assays	Detects histone modifications in circulating nucleosomes	Screening for hepatocellular carcinoma	[183]
	UHPLC‐mass spec	Reversed‐phase separation and tandem mass spectrometry for nucleoside/adduct quantification, with attomolar sensitivity	Pan‐cancer (e.g., leukemia, glioblastoma)	[184]
	SERS (surface‐enhanced Raman spectroscopy)	Antibody‐conjugated Raman tags (e.g., 4‐MBA for H3K27me3) bind to specific PTMs, inducing localized Raman signal enhancement at plasmonic hotspot regions.	Breast and lymphoma profiling	[179, 180, 181]
Noncoding RNAs	RNA sequencing (RNA‐Seq)	Profiles miRNA, lncRNA, and circRNA expression	Biomarker discovery in pancreatic cancer	[174, 185]
	Quantitative PCR (qPCR)	Quantifies specific noncoding RNAs with high sensitivity	Early detection of gastric cancer	[174, 185]
	Microarray analysis	High‐throughput screening of noncoding RNA expression	Identifying miRNAs in breast cancer	[186]
	SERS (surface‐enhanced Raman spectroscopy)	DNA “nanoclaw” probes with complementary miRNA sequences induce plasmon shifts upon hybridization, generating quantifiable Raman peaks for detection	Pancreatic cancer and NSCLC profiling	[179, 180, 181]
	Cyclic voltammetry analysis (electrochemical approach)	Surfactant‐free gold nanostructures enable ultrasensitive (0.012 aM) and selective miR‐9 detection in serum for early lung cancer diagnosis.	Lung cancer (serum miR‐9) detection	[176, 177, 178]

The table shows in detail how liquid biopsy serves in cancer diagnostics together with its functions for treatment and monitoring. Different molecular analysis methods including gene‐specific DNA methylation assessment together with nucleosome profiling and SNP/variant discovery along with RNA‐based tests appear in the table [187]. Two main methods used in cancer research belong to cell‐free and cell‐based approaches. It evaluates liquid biopsy against conventional tissue biopsy showing that liquid biopsy possesses two key benefits through painless sampling and its capacity to examine miRNAs and lncRNAs located in peripheral blood. The ability to detect cancer at early stage together with assessment of prognosis and prediction of treatment response makes this tool highly beneficial for clinical applications [188]. Real‐time liquid biopsy through its analysis of ctDNA and CTCs and tumor proteins and exosomes. The detected analytes enable essential molecular diagnostic assessments along with therapeutic resistance assessments and mutational profile identification and tumor heterogeneity analysis thereby supporting personalized therapy selection [189]. It also describes how selective pressures lead to tumor evolution and metastasis in cancer progression along with relapses. Medical surveillance and individual therapy design becomes possible through liquid biopsy analysis, leading to superior patient results through ongoing patient monitoring and treatment adjustment [190].

## 6 Other Emerging Epigenetic Markers in Liquid Biopsy

Re‐search in liquid biopsies has extended far past the initial discovery of DNA methylation and histone modifications and ncRNA markers. Advancements in epigenetic marker exploration have created extensive opportunities for new investigations about markers employed for disease detection and prognosis assessment and monitoring. Chromatin accessibility has become an important indicator for studying gene expression regulation among recent emerging epigenetic markers. Science has modified ATAC‐seq (assay for transposase‐accessible chromatin with high‐throughput sequencing) to analyze the exposed regions of chromatin in circulating cfDNA through liquid biopsy techniques. Research regions support scientists to understand regulatory landscapes of cancer cells and other diseases states through enabling access to transcription factors and other regulatory proteins [191]. The three‐dimensional DNA structure stands out as a promising research field regarding gene regulation because it affects genetic control mechanisms. The application of chromosome conformation capture (3C) derivatives including 4C, 5C, and Hi‐C helps scientists determine the spatial structure of a genome based on analysis of circulating cfDNA. Liquid biopsy analysis is enhanced through these methods because they show changes in DNA looping and higher order DNA structures that doctors link to cancer development [192]. Scientists have begun to establish DNA fragmentation patterns as essential epigenetic markers. cfDNA fragment size together with end motifs reveals helpful clues regarding disease presence and tissue origin. Tumor‐derived DNA alters its fragment characteristics in unique ways that healthcare providers use to detect cancer as well as track its progression. Disease characterization during liquid biopsy analysis becomes more precise through this specific approach according to research [193]. Histones present in circulating blood serve as a new category of epigenetic biomarkers used in liquid biopsy assessments. Histones that enter the bloodstream exist both independently or in nucleosome complexes while presenting modifications from cellular epigenetics. Studies indicate that circulatory histones demonstrate application in noninvasive diagnostics as disease biomarkers for cancer and sepsis detection [194]. Research dedicated to liquid biopsy now focuses on the investigational value of RNA modifications as sequencing markers. Researchers have discovered that RNA modifications including m6A modifications on mRNA affect multiple aspects of gene regulation. The analysis of particular RNA modifications in circulating RNAs presents opportunities for both disease diagnosis and prognosis which enlarges the potential applications of liquid biopsy platforms [195]. The combination of extra epigenetic markers which include chromatin accessibility, DNA conformation, DNA fragmentation patterns, circulating histones in addition to RNA modifications extends our understanding of how epigenetic processes work in healthy and diseased conditions. The inclusion of these markers into liquid biopsy testing allows researchers and medical personnel to attain better understanding of disease processes and enhance diagnostic abilities and therapeutic method development. Research into these markers continues to enhance liquid biopsy capabilities in creating personalized medicine approaches through noninvasive diagnostics methods for future clinical applications.

## 7 Ongoing Clinical Trials Exploring Liquid Biopsy and Epigenetic Markers in Cancer Diagnosis

Selected clinical trials on epigenetic markers for liquid biopsy in cancer detection and management were summaried in Table 8.

**TABLE 8:** Selected clinical trials on epigenetic markers for liquid biopsy in cancer detection and management.

Trial ID	Title	Objective
NCT04190056 **Location**: USA	Epigenetic priming for immune therapy in ER+ breast cancer	Evaluate the effect of epigenetic modulation using vorinostat on enhancing immune therapy efficacy in ER+ breast cancer. **Cancer type**: Breast cancer **Epigenetic marker**: Histone deacetylation
NCT01845805 **Location**: USA	Phase II trial of CC‐486 in resected pancreatic cancer	Assess the pharmacodynamic effects of CC‐486 on epigenetic and genetic alterations in resected pancreatic cancer tissue. **Cancer type**: Pancreatic cancer **Epigenetic marker**: DNA methylation
NCT04752358 **Location**: USA	Study of ADP‐0055 in advanced solid tumors	Evaluate the safety and efficacy of ADP‐0055, focusing on epigenetic changes in tumor and liquid biopsy samples. **Cancer type**: EGJ cancers **Epigenetic marker**: DNA methylation
NCT04057365 **Location**: USA	Combination of DKN‐01 and nivolumab in advanced biliary tract cancer	Assess the overall response rate of DKN‐01 and nivolumab, exploring circulating biomarkers including epigenetic markers. **Cancer type**: Biliary tract cancer **Epigenetic marker**: Circulating biomarkers
NCT02827838 **Location**: USA	MicroRNA biomarkers in predicting immunotherapy response in solid tumors	Identify circulating microRNA signatures predictive of immunotherapy response in various solid tumors using liquid biopsy. **Cancer type**: HNSCC **Epigenetic marker**: Noncoding RNAs (miRNAs)
NCT04622423 **Location**: France	LiMeT: Liquid biopsy for monitoring epigenetic changes in metastatic tumors	Characterize epigenetic modifications in metastatic tumors through liquid biopsy to monitor treatment response. **Cancer type**: Metastatic tumors **Epigenetic marker**: DNA methylation, histone modification
NCT03199040 **Location**: USA	Neoantigen DNA vaccine alone vs. plus durvalumab in triple negative breast cancer	Evaluate the safety and immunogenicity of a neoantigen DNA vaccine strategy in TNBC patients, assessing epigenetic markers via liquid biopsy. **Cancer type**: Breast cancer **Epigenetic marker**: DNA methylation
NCT02708680 **Location**: USA	ENCORE 301: Entinostat plus exemestane in ER+ metastatic breast cancer	Evaluate the efficacy of entinostat combined with exemestane, analyzing histone acetylation status via liquid biopsy. **Cancer type**: Breast cancer **Epigenetic marker**: Histone acetylation
NCT02666950 **Location**: USA	WEE1 inhibition with AZD1775 in solid tumors	Investigate the effects of WEE1 inhibition on DNA damage response and epigenetic alterations in solid tumors. **Cancer type**: Solid tumors **Epigenetic marker**: DNA methylation
NCT04464174 **Location**: France	MEDOPP253: Liquid biopsy for genomic profiling in advanced solid tumors	Compare genomic DNA data from liquid biopsy with tumor tissue to assess epigenetic alterations in advanced solid tumors. **Cancer type**: Solid tumors **Epigenetic marker**: DNA methylation
NCT03498521 **Location**: USA	FoundationOne liquid CDx in cancer of unknown primary	Utilize liquid biopsy to identify actionable genomic alterations, including epigenetic markers, in cancers of unknown primary origin. **Cancer type**: Cancer of unknown primary origin **Epigenetic marker**: DNA methylation
NCT05060003 **Location**: USA	Signatera MRD test in non‐small–cell lung cancer	Evaluate the use of Signatera ctDNA assay for minimal residual disease detection and epigenetic profiling in NSCLC patients. **Cancer type**: Lung cancer **Epigenetic marker**: DNA methylation
NCT01897571 **Location**: USA	Histone modification analysis in tumor biopsies for cancer diagnosis	Analyze histone modification patterns in tumor biopsies to improve cancer diagnosis accuracy. **Cancer type**: Various cancers **Epigenetic marker**: Histone modification

***Data Source***: All clinical trial information was obtained from ClinicalTrials.gov (https://clinicaltrials.gov).

## 8 Conclusion and Future Prospects

The integration of liquid biopsy into clinical oncology has revolutionized cancer diagnostics by providing a noninvasive, real‐time approach to tumor profiling. Epigenetic markers—particularly DNA methylation, histone modifications, and ncRNAs—have emerged as powerful tools for early cancer detection, prognosis assessment, and therapeutic monitoring. DNA methylation patterns, such as hypermethylation of tumor‐suppressor genes (RASSF1A) and global hypomethylation of oncogenes, serve as stable biomarkers detectable in ctDNA [196]. Furthermore, advancements in NGS and digital PCR (dPCR) have enhanced the sensitivity of methylation profiling, enabling the detection of minimal residual disease (MRD) and relapse prediction [3].

Histone modifications, including acetylation (H3K27ac) and methylation (H3K27me3), play a crucial role in regulating chromatin accessibility and gene expression in cancer. The detection of tumor‐specific histone PTMs in circulating nucleosomes offers a novel avenue for liquid biopsy applications [4]. For instance, EZH2‐mediated H3K27me3 silencing is associated with aggressive tumor behavior and resistance to therapy in breast and prostate cancers [5]. Emerging technologies, such as mass spectrometry and ChIP‐seq adapted for liquid biopsies, are improving the characterization of histone modifications in patient blood samples [6]. Additionally, the development of epigenetic therapies, including HDAC and EZH2 inhibitors, underscores the potential of liquid biopsy in monitoring treatment response and identifying resistance mechanisms [7].

ncRNAs, particularly miRNAs and lncRNAs, have gained prominence as epigenetic biomarkers due to their stability in biofluids and involvement in oncogenic pathways. For example, miR‐21 overexpression is linked to poor prognosis in breast and lung cancer, while miR‐34a functions as a tumor suppressor by regulating p53‐dependent apoptosis [8]. Similarly, lncRNAs such as *MALAT1* and *HOTAIR* are implicated in metastasis and therapy resistance, making them promising targets for intervention [9]. Despite challenges like low abundance and RNA degradation, innovations in RNA‐seq and nanoparticle‐based enrichment techniques are enhancing the reliability of ncRNA detection in liquid biopsies [10]. Future research should focus on multianalyte approaches, combining ncRNAs with DNA methylation and protein biomarkers, to improve diagnostic accuracy.

Looking ahead, the future of liquid biopsy lies in the convergence of multiomics technologies, artificial intelligence (AI), and single‐cell analysis. AI‐driven algorithms can integrate epigenetic, genomic, and proteomic data to predict tumor evolution and optimize personalized treatment strategies [11]. Single‐cell epigenomics, though still in development, hold promise for dissecting tumor heterogeneity and identifying rare metastatic clones [12]. Additionally, large‐scale clinical trials are needed to validate the utility of epigenetic biomarkers in diverse cancer types and populations. As these technologies mature, liquid biopsy is poised to become a standard tool in precision oncology, enabling early detection, dynamic monitoring, and tailored therapeutic interventions.

Looking ahead, the field of liquid biopsy is set to expand significantly. The ongoing refinement of detection technologies, such as enhanced mass spectrometry and NGS, promises to improve the sensitivity and specificity of epigenetic biomarker analysis. This progress will likely usher in a new era of precision oncology where liquid biopsy can be routinely applied not just for advanced cancers but also as a standard screening tool for early cancer detection. Further research and clinical trials are needed to better understand the complex interactions among various epigenetic modifications and their implications for cancer therapy. Innovations in bioinformatics and data analysis will also play a crucial role in interpreting the vast amounts of data generated by liquid biopsies, translating this information into actionable clinical insights. As we continue to unravel the epigenetic mechanisms underlying cancer, liquid biopsy could become a cornerstone of cancer management, transforming the landscape of cancer care by facilitating early detection, real‐time monitoring, and personalized treatment strategies.

## Author Contributions

Debalina Saha, Pritam Kanjilal, Bikram Dhara: Conceptualization, Writing Original Manuscript, Software, Data Curation, Validation, Illustration, Editing. Mandeep Kaur, Soumya V. Menon, Ayash Ashraf, M. Ravi Kumar, Shikha Atteri, Taha Alqahtani: Conceptualization, Writing Original Manuscript, Software, Data Curation, Validation, Daniel Ejim Uti, Bikram Dhara: Conceptualization, Resources, Supervision, Validation, Project Administration, Software, Editing.

## Ethics Statement

The authors have nothing to report.

## Conflicts of Interest

The authors declare no conflicts of interest.

## Data Availability

All used data are within the manuscript.
